# Editorial: Host-microbe interaction and coevolution

**DOI:** 10.3389/fgene.2022.983158

**Published:** 2022-08-30

**Authors:** Wei Huang, Qiang Gao, Kai Fu, Rodrigo Pulgar Tejo, Lucile Maria Floeter-Winter, Alejandro P Gutierrez

**Affiliations:** ^1^ Department of Molecular Microbiology and Immunology, Malaria Research Institute, Johns Hopkins Bloomberg School of Public Health, Baltimore, MD, United States; ^2^ School of Biomedical Science, Hunan University, Changsha, Hunan, China; ^3^ Institute of Molecular Precision Medicine and Hunan Key Laboratory of Molecular Precision Medicine, Xiangya Hospital, Central South University, Changsha, Hunan, China; ^4^ Nutrition and Food Technology Institute, University of Chile, Santiago, Chile; ^5^ Institute of Bioscience, University of São Paulo, São Paulo, Brazil; ^6^ University of Stirling, Stirling, United Kingdom

**Keywords:** pathogen, microbiome, agriculture, host-microbe interaction, alkaloid

Over 100 years ago, Robert Koch established that infectious diseases are caused by microbes. Various studies have shown that different symbiotic and pathogenic microbes could impact host health, including causing diseases. As opposed to studying a single species of microbiota or the host independently, more and more researchers have changed their focus to study how the microbes interact with their hosts, including plants, animals, and fungi.

Studies demonstrating the relationship between microbial communities and their hosts have been emphasized since the last century. Various reports have shown that these microbial communities (microbiota) are dynamic, and their composition depends on host genotype, species, and ontogeny, including diet, habitat, geographical location, and anthropogenic disturbance (Kohl, 2020; Gaete et al., 2021). Importantly, it has been demonstrated that diseases such as diabetes and cancers could affect the human microbiota of the gut and other organs (Iida et al., 2013; Grigorescu and Dumitrascu, 2016). During the last 2 decades, high-throughput sequencing technologies have been widely used to characterize the host’s microbiome (microbiota gene pool), enhancing our understanding of the microbial networks and their interactions with the hosts.

For this Research Topic, we aim to promote knowledge from recent advances in the field of microbiota-host interaction and coevolution and use this information to develop approaches to promote crop, cattle, and human health in the long run. Ten articles in this issue gathered information encompassing recent studies conducted in various hosts including vertebrates like humans, birds, and chickens; invertebrates like honeybees and fig wasps; and plants like *Macleaya cordata*.

It has been demonstrated that these interactions between the microbe and host could maintain benefits for one or for both sides. However, in other cases, this interaction can also be pathogenic, resulting in severe host diseases and even leading to deaths, such as diseases caused by SARS-COV2 and monkeypox virus. Some of these pathogens are also constantly evolving to become resistant to a host’s defense mechanisms. Understanding pathogenesis and the host defenses in the fight against pathogens is, therefore, an urgent topic. Here, four manuscripts focused on human pathogens. Jiang et al. reported that the non-pathogenic *Mycobacterium smegmatis mc251* strain selected against H2O2 stress showed a mutation in the bacterial fur gene, which is responsible for strong ROS resistance and INH sensitivity. They also showed that the redox-related protein Rv1996 showed distinct phenotypes under different physiological conditions among different species of *Mycobacterium* species. This may somehow explain why despite having similar virulence factors and signaling transduction systems, *Mycobacterium tuberculosis* (TB) and *Mycobacterium smegmatis mc251* strains showed different virulence against their hosts (Jiang et al.). The phenomenon suggests that *M. smegmatis mc251* could be a suitable candidate vector for a vaccine for TB. Shi et al. investigated the polymorphism of PvDBP (PvDBP-II) in the human pathogen *Plasmodium vivax* from the China-Myanmar Border of Yunnan Province, China, which could be targeted to generate vaccines against malaria (Shi et al.). Another important study conducted by Lv et al. found that the two-component system QseBC from Hypervirulent *Klebsiella pneumonia* contributes to the virulence of the human (Lv et al.). This research provided new insights into the functional importance of QseBC in regulating the virulence of hvKP. Wu et al. collected seven *Cyclophyllidea* specimens from rodents in Qinghai-Tibet Plateau (QTP) and found that some of them might affect human health (Wu et al.).

The microbiota is also crucial to the agricultural industry and environmental protection. Three research articles are focused on this area. Yang et al. investigated the microbiota in the asexual hybridization queens (AHQs) of honeybees (Yang et al.). This research revealed that genetic background rather than environmental factors is dominant in shaping the gut microbiota. Li et al. focused on the microbiota in the fig wasp genus *Ceratosolen* (Li et al.). They found that the endosymbiotic *Wolbachia* carried by fig wasps led to a decrease in bacterial diversity. Huang et al. showed that subchronic copper exposure induced metabolic disorders in chickens by disrupting the microbial community, which may induce metabolic disorders in chickens (Huang et al.). This research will guide the chicken farm to optimize the usage of copper.

Two research articles focused on the microbiota diversity in birds from the Tibetan wetland and an endangered bird *Nipponia Nippon* (Zhu et al.; Bo et al.). The authors collected crucial data on microbes correlated with those birds’ development and growth.

The final report revealed the consequences of plant niches and alkaloids on the bacterial community, which could help us to better understand microbe-plant interactions. In this study, high-throughput gene sequencing was used to investigate the composition and abundance of bacteria from the rhizospheric soil and different parts of *Macleaya cordata*, which is well known as traditional antibacterial medicine (Kosina et al., 2010; Sai et al., 2015). *Sphingomonas* were observed to have a significant positive correlation with allocryptopine and protopine, while allocryptopine, protopine, chelerythrine, and sanguinarine showed a negative correlation with the microbiota of stems. These results indicated that bacterial abundance was the highest in the rhizospheric soil and there was a higher diversity of endophytic microorganisms with genes related to alkaloid synthesis indicating a mutual benefit for both the host and the microbe (Lei et al.). This is a good example of host-microbe interaction and evolution. The *Macleaya cordata* secretes alkaloid which shapes the microbiota, while, the microbiota also affects the growth and alkaloid production of *Macleaya cordata.*

Overall, this Research Topic highlights the role of microbiota in human pathogens as well as in agricultural animals and beneficial insects, all of which are of great significance to guide clinical applications, agriculture, and wild animal protection. The host-microbe interaction studies contribute to a better understanding of the role of microbiota in organism development and disease pathology. Understanding the interaction between the microbe and the host would guide us to increase the benefits and reduce harm from microbiota. We also noticed six manuscripts in this issue used high-throughput sequencing technologies to study different organisms. There is no doubt the microbiome analysis will provide fundamental data for further study of microbiota networks and host-microbe interaction. In general, high-throughput microbiome sequencing generates a flood of new data, but how to quickly mine valuable data and use the data to guide us to improve human health and environmental protection is still a challenge for future studies.
